# Novel poly (*N*-methacryloyl-L-alanine acid) grafted chitosan microspheres based solid-phase extraction coupled with ICP-MS for simultaneous detection of trace metal elements in food

**DOI:** 10.1016/j.fochx.2023.100926

**Published:** 2023-10-15

**Authors:** Yichuan Cao, Jiaxing Qin, Zheng Su, Lin Cai, Guozhen Fang, Shuo Wang

**Affiliations:** a State Key Laboratory of Food Nutrition and Safety, Tianjin University of Science and Technology, Tianjin 300457, China; b Research Center of Food Science and Human Health, School of Medicine, Nankai University, Tianjin 300071, China

**Keywords:** *N*-methacryloyl-L-alanine acid, Tartaric acid-crosslinked chitosan microspheres, Metal ions, Solid phase extraction, ICP-MS

## Abstract

Poly (*N*-methacryloyl-L-alanine acid) grafted tartaric acid-crosslinked chitosan microspheres (PNMA-TACS) were successfully synthesized and employed as a novel adsorbent for the separation and enrichment of metal ions in the food system. PNMA-TACS microspheres-based solid phase extraction (SPE) was coupled with ICP-MS for accurate quantification of trace V(V), Cr(III), As(III), Pb(II), Cd(II) and Cu(II). The obtained PNMA-TACS microspheres were characterized, and parameters influencing the method were optimized. Under optimal conditions, the calibration curves for Cu(II) and V(V) were linear within 0.01–30 μg L^−1^, the linear ranges of Cr(III), As(III), Pb(II) and Cd(II) were 0.01–15 μg L^−1^, and the detection limit of the developed approach was 1.1–3.7 ng L^−1^. The results were consistent with the consensus values of method validation implemented by two standards. Moreover, standard addition recovery experiments were performed in rice and milk powder, which achieved satisfactory recovery of 86.1–103.5%.

## Highlights

• Novel adsorbent of (*N*-methacryloyl-l-alanine acid) grafted chitosan microspheres was fabricated.
• The simultaneous detection of V(V), Cr(III), Cu(II), Cd(II), As(III) and Pb(II) was realized in food samples.
• ICP-MS assisted with SPE method based on the prepared adsorbent was established.
• The method exhibited excellent high recoveries in the elements analysis of practical samples.

## 1 Introduction

Much emphasis has been placed on heavy metal scavenging from polluted water over the years, but poor removal efficiency has resulted in trace levels of toxic ions being discharged into the environment (Alizadehgiashi et al., 2018, Sun et al., 2017). Excessive heavy metals in the environment result in a variety of ecosystem problems, which can accumulate in aquatic organisms migrate into soil, and further transfer to the body via food chains, posing a serious threat to human health (Saravaia et al., 2018, Zhang et al., 2017). Therefore, a new detection method used for the simultaneous detection of multiple elements with good sensitivity and accuracy is of great importance for food safety detection.

Various instrumental methods such as atomic absorption spectroscopy (Ogunfowokan et al., 2019), ICP-OES (Hong et al., 2019) and ICP-MS (Shaheen et al., 2021, Itoh et al., 2018) have been widely used for detecting trace metal elements in real samples. The direct analysis of real samples by these techniques is limited by matrix interference and the inability to measure extremely low concentrations of analytes below the instrumental limit detection (He et al., 2017). Therefore, it is essential to select an appropriate sample pretreatment method before analysis. Of all metal ions treatment techniques, solid-phase extraction (SPE) is a preferred technique for elemental separation due to the fast phase separation, simple operation and higher enrichment factor (Sun et al., 2018). Since the adsorption materials play a crucial role in SPE, several adsorbents have been employed in SPE, such as active carbon (Shin et al., 2022), functionalized silica gel (Imamoglu, 2023); agarose (Pourmand et al., 2015); and chitosan (CS) and its derivatives (Djerahov et al., 2016).

Chitosan (CS) is an interesting natural polymer with abundant amino and hydroxyl groups, which is regarded as a sustainable adsorbent for chelating metal ions (Maity and Ray, 2018). Several chemical modification techniques such as crosslinking and grafting can improve mechanical strength and chemical stability, expanding the potential application of this biopolymer. The most common cross-linkers of chitosan are aldehydes (Xu et al., 2013), epoxides, organic acid and other reagents (Saeedi et al., 2022). Organic acids, effective crosslinking agents, can introduce plenty of oxygen atoms to provide compensation for adsorption sites via ionic or covalent bonding between the acid and hydroxyl group or amino group. Polycarboxylic acids allow the formation of bridges between polymeric chains, similar to sodium tripolyphosphate, such as malic acid (Wang et al., 2018), oxalic acid (Mi et al., 2015); succinic acid (Gabriele et al., 2021) and citric acid (Bagheri et al., 2015), which have at least two reactive carboxyl groups. Various chitosan derivates have been used to improve the adsorption ability of chitosan by grafting vinyl monomers with new function groups on the crosslinked chitosan backbone, such as acrylic acid (Xu et al., 2014); methacrylic acid (Huang et al., 2013), 2-acrylamido-2-methylpropane sulfonic acid (Xu et al., 2018); itaconic acid (Kyzas et al., 2014) and malic acid (Yu et al., 2017). It is well-established that amino acids are suitable metal-scavenger molecules for various electron donor atoms, including oxygen and nitrogen, which can be incorporated into polymers through free radical polymerization of vinyl monomer carrying amino acid residues. No study has hitherto reported adsorbing metal ions by tartaric acid-chitosan microspheres with an alanine graft.

**Scheme 1:**
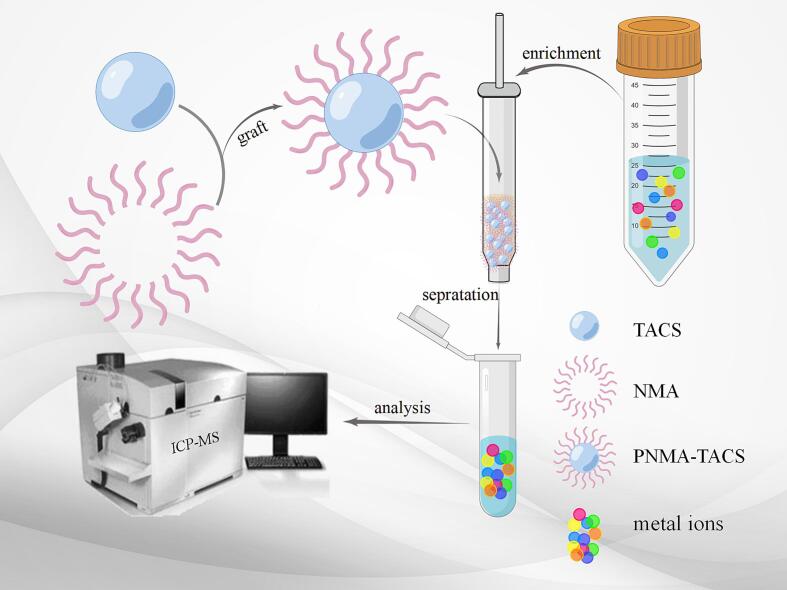
Schematic illustration in the synthesis process of PNMA-MACS microspheres and the detection of trace elements by ICP-MS.

In this work, we describe a novel method for simultaneously detecting six metal ions using poly (*N*-methacryloyl-L-alanine acid) grafted and tartaric acid-crosslinked chitosan microspheres (PNMA-TACS) as solid phase extraction adsorbent for preconcentration and separation of heavy metal ions in SPE-ICP-MS analysis. As shown in Scheme 1, the experiment employed tartaric acid (TA) as a crosslinking agent to facilitate the crosslinking of CS through the formation of ionic or covalent bonds between carboxyl and hydroxyl or amino groups. The obtained chitosan microspheres (TACS) with amino groups exhibited excellent net-like architecture. *N*-methacryloyl-L-alanine acid (NMA) was obtained by coupling methacryloyl chloride with alanine acid to prepare amino-acid based polymers. Subsequently, by employing the addition reaction between amino groups and double bonds, NMA was grafted onto the surface of chitosan microspheres to synthesize alanine modified chitosan microspheres. The prepared PNMA-TACS microspheres exhibited a larger specific surface area, and more adsorption sites and groups, improving the adsorption ability of metal elements. Thus, the proposed SPE-ICP-MS method based on PNMA-TACS displayed good application potential.

## 2 Experiment

### 2.1 Materials and reagents

Chitosan, tartaric acid, 4-dimethylaminopyridine (DMAP), sodium hydroxide, L-alanine, 1-(3-Dimethylaminopropyl)-3-ethylcarbodiimide hydrochloride (EDC), methacryloyl chloride and anhydrous sodium sulfate were obtained from Macklin Biochemical Co., Ltd. (Shanghai, China). Ammonium persulphate (APS), sodium hydrogen sulfite, ethanol, hydrochloride, ammonium solution and ethyl acetate were purchased from Sinopharm Chemical Reagent Co., Ltd. (Tianjin, China). The metal standard solutions (10 mg L^−1^) were supplied by Agilent Technologies Trading Co., Ltd. (Shanghai, China), and reference materials (scallop and rice) from the Center of National Standard Reference Material of China.

### 2.2 Characteristics

Morphology and microstructure of TACS microspheres and PNMA-TACS microspheres were obtained with an Apreo scanning electron microscope (SEM) (FEI, USA) under 8 k, 15 k magnification. The Fourier transform infrared spectra of the synthesized materials were recorded on Bruker Tensor27 spectrometer (BRUKER, Germany) in the range of 4000–400 cm^−1^. The pore volume, pore size and specific surface area of TACS and PNMA-TACS microspheres were acquired using an Autosorb-1-MP (QUANTACHROME, USA). The TG curves were accomplished on a Q50 thermogravimetric analyzer (TA, USA) at temperatures from 30°C to 600°C with a 20°C min^−1^ heating rate. The number of elements was measured by ICP-MS 7500CX (AGLIENT, USA), and MARS6 (CEM, USA) was used as a microwave digestion system.

### 2.3 Preparation of PNMA-TACS microspheres

#### 2.3.1 Preparation of TACS microspheres

TACS microspheres were synthesized using a reversed-phase emulsification method previously reported (Wang et al., 2018). Specifically, the water phase (solution A) was obtained by dissolving CS powder (0.25 g) in acetic acid solution (12.5 mL, 2%, v/v). Tartaric acid (TA 0.22 g), EDC (0.18 g), and DMAP (0.18 g) were dispersed in 4 mL deionized water under magnetic stirring, followed by adjusting the pH to 5.0 to obtain the cross-linking agent (solution B) for further use. Then, solution A was added into the flask containing 2 mL span-80 and 40 mL liquid paraffin, followed by the slow addition of solution B. The resultant TACS microspheres were obtained after stirring for a further 3 h at 60°C.

#### 2.3.2 Preparation of NMA monomers

The method for the synthesizing of *N*-methacryloyl-L-alanine acid was reported in detail in a previous study (Xie et al., 2014). Briefly, L-alanine (2.32 g, 26 mmol) was dissolved in a flask containing 30 mL 1.5 mol L^−1^ NaOH aqueous solutions with continuously stirring until the L-alanine was dispersed completely. Subsequently, methacryloyl chloride (2.5 mL, 26 mmol) was dropwise added to the solution at 0–5°C under N_2_ surroundings. The mixture was continuously stirred for 2 h, and then the reaction solution was heated up to ambient temperature and stirred for another 1 h. Furthermore, 6 mol L^−1^ HCl aqueous solution was used to acidify mixture to a pH of 2–3, then extracted with ethyl acetate five times. The organic phase was dried over anhydrous Na_2_SO_4_ and evaporated to obtain crude samples. Monomer NMA was acquired by recrystallization from ethanol.

#### 2.3.3 Preparation of NMA grafted TACS microspheres

NMA was grafted onto TACS microsphere surfaces through free-radical polymerization (Ge and Hua, 2016). TACS microspheres (1.0 g), APS (0.18 g), SBS (0.06 g), and deionized water (50 mL) were mixed to obtain a homogeneous solution. Then, NMA (5 mL 1.5 g) was added into the mixtures under magnetic stirring at 60°C. After stirring for 2 h, PNMA-TACS microspheres were collected after drying to constant weight.

### 2.4 Pre-concentration by SPE

20 mg of the PNMA-TACS microspheres were packed into the PTFE extraction column and activated by 1.0 mol L^−1^ HNO_3_, then impurities were removed by deionized water. 20 mL of the sample solution containing 1.0 µg L^−1^ V(V), As(III), Cr(III), Cd(II), Pb(II) and Cu(II) was passed through the SPE column at a flow rate of 1.5 mL min^−1^. The retained metals on PNMA-TACS microspheres were eluted in the case of 2.5 mL HNO_3_ (0.5 mol L^−1^). The metal concentrations were detected by ICP-MS and the samples were tested in three parallel measurements.

### 2.5 Samples application

The method was validated by using two standards of scallop (GBW 10024) and rice (GBW10043), and the practicality was proved by the recovery tests in digested rice and milk powder (Qin et al., 2021). The acid digestion process using HNO_3_ and H_2_O_2_ with microwave-assisted digestion was conducted. Each sample, weighing precisely 0.2 g, was placed in a PTFE digestion tank. Subsequently, 5 mL of nitric acid (65% w/w) was added in a fume hood, and the samples were sealed and left overnight. Following the addition of 2 mL of 35% H_2_O_2_, all samples were subjected to microwave digestion as per the predetermined procedure. After the reaction, the pH was adjusted to pH = 6 using ammonia solution, and each sample was filtered through a 0.22 μm pore size membrane filter.

## 3 Results and discussion

### 3.1 Characterization of PNMA-TACS microspheres

**Fig. 1:**
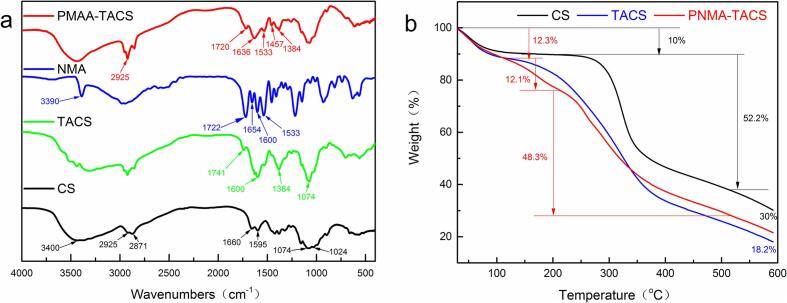
The FTIR spectrums of synthesis process of PNMA-TACS microspheres (a) and the TG curves of PNMA-TACS microspheres in the synthesis process (b).

The synthesis of PNMA-TACS microspheres was preliminarily validated by FTIR spectroscopy. The FTIR spectra of PNMA-TACS, NMA, TACS and raw chitosan were shown in Fig. 1a. For the CS powder, the main characteristic bands were as follows: the peak around 3400 cm^−1^ represented the stretching vibrations of –OH, –NH and intermolecular hydrogen bonding, the peaks at 2925 and 2871 cm^−1^ were assigned to asymmetric and symmetric C—H stretching vibrations, 1660 and 1595 cm^−1^ were respectively ascribed to amide I and –NH_2_ bending vibration due to the incomplete deacetylation of chitosan. Peaks at 1074 and 1024 cm^−1^ were respectively related to the stretching vibrations of C—O on C3-OH and C6-OH. In TACS microspheres spectrum, along with the decreased peak at 1024 cm^−1^, the emergence of a pronounced new peak at 1741 cm^−1^ was due to the bending vibration of O-C=O formed by amino and carboxyl via an esterification reaction. Besides, the 1384 cm^−1^ peak belonged to the symmetric stretching vibration of carbonyl in free carboxyl (Senna et al., 2014); which implied the successful cross-link between CS and TA. Moreover, for PNMA-TACS microspheres, a shoulder-like band emerged at 1720 cm^−1^ due to the carboxyl groups. The peaks appeared at 2925 cm^−1^ (C—H stretching vibrations), 1636 cm^−1^ (amide I) and 1457 cm^−1^ (C—N stretching vibrations) deducing that NMA was successfully grafted on the TACS microsphere surface. Simultaneously, the enhanced absorption at 1533 cm^−1^ was attributed to the bending vibration of –NH formed in free-radical polymerization with the involvement of –NH_2_ and –C=C– (Liu et al., 2015, Vakili et al., 2019). Thus, the above findings initially demonstrated the successful preparation of PNMA-TACS microspheres.

The thermal properties of materials during the synthesis process were investigated by TG curves in (Fig. 1b). The initial mass loss at the range of 30–15°C was attributed to moisture evaporation, while a rapid weight loss of 60.1% was observed from 230 to 500°C, indicating the thermal degradation of CS chain. Compared with the CS, the thermogravimetric curve of TACS with less residual (18.2%) at 600°C was caused by incorporating cross-linking reagent. Besides, the lower onset temperature (120°C) and a lower rate of weight loss in the second stage were ascribed to the decrease of intermolecular hydrogen bonds. These findings proved that CS was cross-linked by tartaric acid. For PNMA-TACS, there were three decomposition stages at 30–600°C. The weight loss (12.3%) below 100°C was involved by the evaporation of water. There was about 12.1% weight loss from 110 to 210°C due to the decomposition of small monomer molecules in the second stage (Liu et al., 2015). The final weight loss was associated with the degradation of CS side chains and backbone. Such changes might illustrate that the grafting reaction has occurred in the synthesis process.

**Fig. 2:**
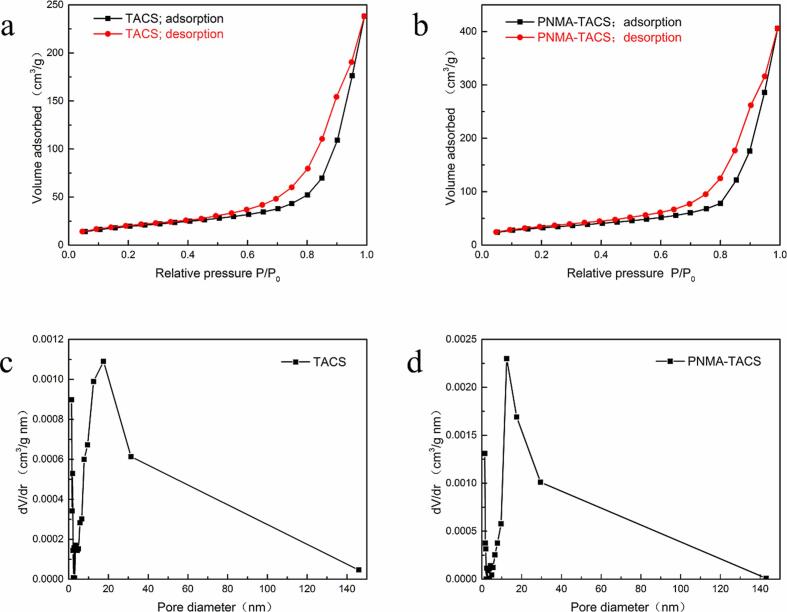
The N_2_ isotherms and pore size distribution curves of TACS (a, c) and PNMA-TACS microspheres (b, d).

The microstructure of TACS and PNMA-TACS microspheres was determined by N_2_ adsorption analysis at 77 K. The adsorption isotherms of these materials (Fig. 2a-b) exhibited type IV characteristics with hysteresis loops and convex upward trends in the low relative pressure (P/P_0_) region according to IUPAC classification, implying that both microspheres were typical mesoporous materials. After grafting the NMA on the surface of TACS microspheres, the Brunauer-Emmett-Teller (BET) surface area of PNMA-TACS (113.24 m^2^ g^−1^) was much larger than that of TACS (68.93 m^2^ g^−1^), enhancing the adsorption of metal ions. The pore size distribution curves of TACS and PNMA-TACS calculated using the Barrett-Joyner-Halenda (BJH) method was shown in Fig. 2c-d. The average pore size of TACS and PNMA-TACS was 21.43 nm and 22.23 nm, respectively. The total pore volume of PNMA-TACS was found to be 0.63 cm^3^ g^−1^, which is 1.7-fold higher than that of TACS (0.37 cm^3^ g^−1^). Our results suggest that the higher total pore volume and BET surface area after grafting were beneficial to the adsorption of metal ions.

**Fig. 3:**
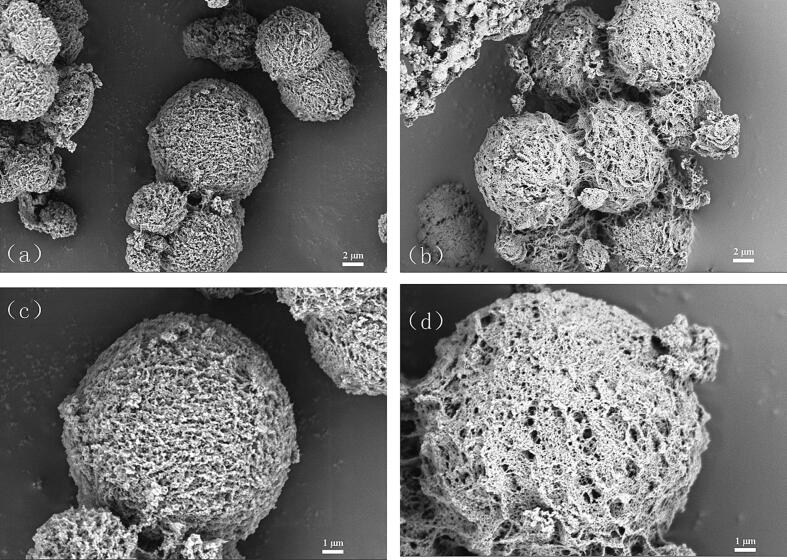
The scanning electron microscopy (SEM) micrographs of TACS (a, c) and PNMA-TACS microspheres (b, d).

In the SEM mapping (Fig. 3), the surface morphologies of TACS and PNMA-TACS microspheres were observed as spherical or nearly spherical. TACS microspheres exhibited rough morphologies with striations and unevenly distributed pits, meanwhile abundant exposed amino groups were beneficial to the grafting reaction. Compared to TACS microspheres, the surface of PNMA-TACS microspheres seemed to be rougher with slight protuberances and porous. Consistent with BET results, these changes were substantially associated with the grafting of PNMA, which provided more adsorption sites for metal ions.

### 3.2 Optimization of the experimental parameters

The main parameters in SPE were investigated to achieve good sensitivity, including the pH and flow rate of sample loading, the dose of adsorbent as well as the volume and concentration of eluent. The analyte concentrations of the sample and elute solutions were detected by ICP-MS, respectively, which was used to define recovery. The high recoveries (>85%) of target ions promoted the performance of the PNMA-TACS-SPE column.

#### 3.2.1 Effect of the solution pH

**Fig. 4:**
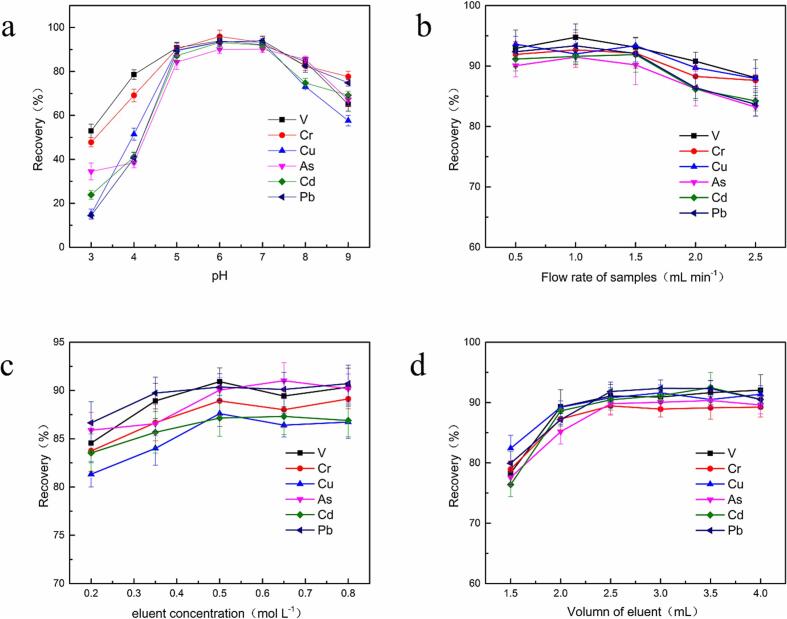
The effect of the SPE parameters: (a) the pH of the sample solution; (b) the flow rate of sample loading; eluent concentration (c) and volume (d).

It is widely acknowledged that the initial sampling pH played a crucial role in extraction efficiency of the adsorption, which exhibited partial protonation of the active groups and the competition of H^+^ with metal ions occupying the active sites on the surface in an acidic environment or precipitating as metal hydroxide in alkali solution (Vakili et al., 2019). Thus, the impacts of sampling pH on the recoveries of adsorbents for each analyte were investigated in the range of 3.0–9.0, which was adjusted by adding HNO_3_ and NH_3_·H_2_O. As shown in Fig. 4a, the recoveries of each analyte exhibited an upward trend with the increase of sampling pH at 3.0–6.0, and the best recovery exceeding 85% was accessed at pH 6.0–7.0. When the initial pH was higher than 7.0, metal hydroxides would form to decrease the recoveries of the analyte. Therefore, the initial sample solution pH was regulated to 6.0.

#### 3.2.2 Effect of adsorbent dose

To select an appropriate amount of PNMA-TACS microspheres in the SPE column, columns packed with PNMA-TACS microspheres of 20, 30 and 40 mg were used to investigate the effect of adsorbent dose on the extraction of target ions. As shown in Fig. S1, there was no significant difference in recoveries for each analyte by using columns of the varied adsorbent doses. Aimed to minimize the cost of extraction per sample and extraction column pressure, 20 mg was considered the adsorbent dose for the following experiments.

#### 3.2.3 Effect of the flow rate

The effect of the sample loading flow rate on SPE production was investigated to improve the adsorption effect by controlling the adsorption time. 20 mL target sample solution was passed through the column under the increasing flow rate from 0.5 to 1.5 mL min^−1^. As indicated in Fig. 4b, the recoveries of each analyte were slightly decreased at flow rates higher than 1.5 mL min^−1^. Meanwhile, in order to decrease the extraction time, the flow rate was set at 1.5 mL min^−1^ for subsequent procedures.

#### 3.2.4 Effect of eluent concentration

During the elution process, HNO_3_ was an effective reagent to strip the extracted target ions from the adsorbent, which was selected as the eluent for ICP-MS. Various concentrations of HNO_3_ at 0.2–0.8 mol L^−1^ were examined for desorption of extracted analytes on the column. Fig. 4c indicated that the target ions could be eluted quantitatively within the range of 0.5–0.8 mol L^−1^, so 0.5 mol L^−1^ was identified as the optimum concentration of elution.

#### 3.2.5 Effect of eluent volume

The volume of eluent was varied within the range of 1.5–4.0 mL to examine the influence of eluent volume. As seen in Fig. 4d, the recoveries of the analytes improved as the increase of eluent volume and reached equilibrium when the volume exceeded 3 mL. Thus, 3.0 mL of eluent was used for SPE desorption.

### 3.3 Evaluation of interference

The mineral ions usually exit in water and biological samples and may interfere with the determination of target ions by ICP-MS. Typical coexisting ions K^+^, Na^+^, Mg^2+^, Ca^2+^, Fe^3+^, Ni^2+^ and Zn^2+^ were added to the solution to investigate the anti-interference ability of SPE-ICP-MS. The tolerance limit was estimated based on the examined ions recoveries no less than 80%, and the presence of 5 mg L^−1^ K^+^ and Na^+^, 5 mg L^−1^ Mg^2+^ and Ca^2+^ and 0.5 mg L^-1^ Fe^3+^, Ni^2+^ and Zn^2+^ had no obvious effect on the determination of all analytes (Table S1). Therefore, the proposed SPE-ICP-MS method with the assistance of PNMA-TACS microspheres might have an excellent anti-interference ability. The concentration ratio between target ions the coexisting ions and the coexisting ions reached 500–5000, indicating that the approach could be employed to detect six different metal ions in food products.

### 3.4 Analytical performance

The linear range and detection limit were examined to evaluate the analytical performance of this method under the investigated conditions. A series of sample solutions with different concentrations of 0.05, 0.1, 0.5, 1.0, 5.0, 10, 15 and 30 μg L^−1^ were prepared to obtain the calibration curves of the intensity of metal ions after extraction. As shown in Table S2, the calibration curve for Cu(II) and V(V) was linear within the range of 0.01–30 μg L^−1^ and the linear range for Cr(III), Cd(II), As(III) and Pb(II) was 0.01–15 μg L^−1^. According to three times the standard deviation of the blank, the limit of detection (LOD) of analytes was 2.6, 1.1, 3.7, 2.3, 1.9 and 2.1 ng L^−1^, respectively. In addition, the recyclability of the prepared adsorbents was investigated. The result showed that after repeated use 5 times, the recovery rate with a slight decrease can remain above 80%, demonstrating the stability and recycling ability of the PNMA-TACS microspheres.

### 3.5 Sample analysis

To check the reliability of this method, two standard reference materials (GBW10043 Rice and GBW 10024 Scallop) were analyzed using the SPE-ICP-MS procedure. It can be seen from Table S3, the analytical results were coincident with certified values, demonstrating that the proposed method was dependable. The recovery assay was conducted in rice and dried milk (Table S4) and exhibited satisfactory recoveries of all target ions ranging from 86.1 to 103.5%, which further validated the accuracy of this method. Therefore, these data indicated that the proposed SPE-ICP-MS method was able to analyze the six target ions in real samples.

### 3.6 Comparison with other adsorbents

The performance of the prepared PNMA-TACS microspheres was compared with the other modified materials reported recently (Sun et al., 2018, Pourmand et al., 2015, Nyaba and Nomngongo, 2020, Peng et al., 2015, Habila, 2016), and the results were summarized in Table 1. Obviously, the developed method was able to simultaneously detect six metal species in food samples. Moreover, the proposed method exhibited a higher sensitivity with lower LODs and RSD parameters than other reported methods. Therefore, the developed method combining SPE-ICP-MS with PNMA-TACS microspheres was successfully applied in the simultaneous determination of six metal ions in real food samples.

**Table 1:** Comparison with other adsorbents in term of analytical performance.

Material	Element	LODs	RSD	Sample	Ref
AAPTS	Ni, Cu, Ga, Cd, Pb, Bi	9–41	1.78–4.73	Milk	(Sun et al., 2018)
agarose-g-PMMA	Cd, Ni, Cu, Zn	0.6–1.8	2.1–4.9	Vegetable, water	(Pourmand et al., 2015)
Mg/Al-LDH@CNTs	As, Cd, Co Cr, Pb, Sb, Tl	60–150	1.9–4.5	Water, spinach	(Nyaba and Nomngongo, 2020)
AAPTS- MWCNTs	As, Cr, Se	15, 38, 16	7.4, 2.4, 6.2	Water	(Peng et al., 2015)
Fe_3_O_4_@SiO_2_@TiO_2_	Cu, Zn, Cd, Pb	4.1–8.2	<10	Tap water	(Habila, 2016)
AM-AC	Ni	352	5.67	Vegetable	(Ebraiumi, 2017)
Fe_3_O_4_@4PhMT-GO	Al	1.5	2.5	Wastewater	(Shirkhanloo et al., 2021)
Amberlite-8-HQ	Mn, Cu, Co, Ni	24–58	1.08–1.66	Groundwater	(Labban et al., 2020)
MWCNTs-BPEI	As	53.7	5.23	Water	(Letsoalo et al., 2019)
PNMA-TACS	V, Cr, Cu, As, Cd, Pb	1.1–3.7	1.5–3.0	Rice, milk powder	This work

## 4 Conclusions

The PNMA-TACS microspheres were prepared as a novel solid phase extractor to establish a sensitive and accurate method for simultaneous determination of six metal ions in real samples. Here, the successfully modified TACS microspheres by alanine exhibited larger surface areas and rougher surfaces, providing abundant adsorption sites for enrichment and isolation of the analytes. The proposed method with high anti-interference ability exhibited excellent sample recovery, wide linear calibration range, and low limit of quantitation. Therefore, the SPE-ICP-MS method based on the PNMA-TACS was expected to be applied for detecting trace V(V), Cr(III), As(III), Cd(II), Pb(II) and Cu(II) in food items.

## CRediT authorship contribution statement

**Yichuan Cao:** Conceptualization, Methodology, Formal analysis, Investigation, Data curation, Writing – original draft, Writing – review & editing, Visualization. **Jiaxing Qin:** Conceptualization, Methodology, Formal analysis, Investigation, Validation, Data curation, Writing – original draft, Visualization. **Zheng Su:** Project administration, Data curation. **Lin Cai:** Resources, Data curation, Project administration. **Guozhen Fang:** Resources, Supervision, Project administration, Funding acquisition. **Shuo Wang:** Funding acquisition.

## Declaration of Competing Interest

The authors declare that they have no known competing financial interests or personal relationships that could have appeared to influence the work reported in this paper.

## Data Availability

Data will be made available on request.
