# Supplementary material for: Novel poly (N-methacryloyl-L-alanine acid) grafted chitosan microspheres based solid-phase extraction coupled with ICP-MS for simultaneous detection of trace metal elements in food
Source: Food Chem X. 2023 Oct 15;20:100926. doi: 10.1016/j.fochx.2023.100926 (PMC10739841; doi:10.1016/j.fochx.2023.100926)
Supplement: Supplementary data 1 [file mmc1.docx]

**Supplementary material**

**

**

**Fig. S1** The effect of adsorbent doses in SPE procedures

Table S1 Tolerance limit of the proposed method in the presence of coexisting ions

| Coexisting ions | Tolerance limit (mg L^−1^) | Concentration ratio |
| --- | --- | --- |
| K(I), Na(I) | 5 | 5000 |
| Mg(II), Ca(II) | 5 | 5000 |
| Fe(III), Ni(II), Zn(II) | 0.5 | 500 |

Table S2 The analytical performance of proposed method

| Element | Line range (μg L^-1^) | R^2^ | LODs (ng L^-1^) | RSD (%, n=11) |
| --- | --- | --- | --- | --- |
| V | 0.01-30 | 0.9983 | 2.6 | 1.5 |
| Cr | 0.01-15 | 0.9973 | 1.1 | 1.8 |
| Cu | 0.01-30 | 0.9930 | 3.7 | 2.4 |
| As | 0.01-15 | 0.9919 | 2.3 | 2.0 |
| Cd | 0.01-15 | 0.9939 | 1.9 | 1.9 |
| Pb | 0.01-15 | 0.9983 | 2.1 | 3.0 |

Table S3 The analytical results of two standard reference materials (mean ± SD, n=3)

| Standard reference materials | Target elements | certified values (μg g^-1^) | Found (μg g^-1^) |
| --- | --- | --- | --- |
| GBW10043 | V | 0.03 | 0.03405 ± 0.00481 |
|  | Cr | 0.14 ± 0.025 | 0.15800 ± 0.01020 |
|  | Cu | 1.7 ± 0.1 | 1.65133 ± 0.09477 |
|  | As | 0.114 ± 0.018 | 0.11638 ± 0.00135 |
|  | Cd | 0.012 ± 0.003 | 0.01017 ± 0.00606 |
|  | Pb | 0.075 ± 0.025 | 0.08775 ± 0.02291 |
| GBW10024 | V | 0.36 ± 0.10 | 0.36706 ± 0.01523 |
|  | Cr | 0.28 ± 0.07 | 0.22500 ± 0.00436 |
|  | Cu | 1.34 ± 0.18 | 1.21982 ± 0.00948 |
|  | As | 3.6 ± 0.6 | 3.54798 ± 0.29434 |
|  | Cd | 0.00106 ± 0.00010 | 0.00098 ± 0.00014 |
|  | Pb | 0.120 | 0.11836 ± 0.00597 |

Table S4 The analytical results of practical sample (mean ± SD, n=3)

| Samples | Target elements | Added (μg g^-1^) | Found (μg g^-1^) | Recovery (%) |
| --- | --- | --- | --- | --- |
| Rice | V | 0 | 0.0436 ± 0.0044 |  |
|  |  | 0.05 | 0.0942 ± 0.0031 | 101.2 |
|  |  | 0.10 | 0.1407 ± 0.0017 | 97.1 |
|  | Cr | 0 | 0.5281 ± 0.0014 |  |
|  |  | 0.1 | 0.6097 ± 0.0081 | 81.6 |
|  |  | 0.5 | 1.0019 ± 0.0073 | 94.8 |
|  | Cu | 0 | 4.8575 ± 0.1015 |  |
|  |  | 0.5 | 5.2869 ±0.0944 | 85.9 |
|  |  | 1.0 | 5.7915 ± 0.1231 | 93.4 |
|  | As | 0 | ND^a^ |  |
|  |  | 0.05 | 0.0488 ±0.0026 | 97.6 |
|  |  | 0.10 | 0.1035 ± 0.0045 | 103.5 |
|  | Cd | 0 | ND^a^ |  |
|  |  | 0.05 | 0.0432 ± 0.0009 | 86.4 |
|  |  | 0.10 | 0.0943 ± 0.0024 | 94.3 |
|  | Pb | 0 | 0.4210 ± 0.0170 |  |
|  |  | 0.10 | 0.5174 ± 0.0153 | 96.4 |
|  |  | 0.50 | 0.9056 ± 0.0237 | 96.9 |
| Milk powder | V | 0 | ND^a^ |  |
|  |  | 0.05 | 0.0472 ± 0.0035 | 94.4 |
|  |  | 0.10 | 0.0972 ± 0.0043 | 97.2 |
|  | Cr | 0 | ND^a^ |  |
|  |  | 0.05 | 0.0458 ± 0.0026 | 91.6 |
|  |  | 0.10 | 0.0953 ± 0.0019 | 95.3 |
|  | Cu | 0 | 0.0196 ± 0.0089 |  |
|  |  | 0.05 | 0.0702 ± 0.0034 | 101.2 |
|  |  | 0.10 | 0.1175 ± 0.0055 | 97.9 |
|  | As | 0 | ND^a^ |  |
|  |  | 0.05 | 0.0422 ± 0.0046 | 84.4 |
|  |  | 0.10 | 0.0896 ± 0.0073 | 89.6 |
|  | Cd | 0 | 0.0280 ± 0.0017 |  |
|  |  | 0.05 | 0.0762 ± 0.0064 | 96.4 |
|  |  | 0.10 | 0.1243 ± 0.0049 | 96.3 |
|  | Pb | 0 | ND^a^ |  |
|  |  | 0.05 | 0.0483 ± 0.0039 | 96.6 |
|  |  | 0.10 | 0.9349 ± 0.0075 | 93.5 |

^a^ Not Detectable
